# Sex Differences, Estrogen Metabolism and Signaling in the Development of Pulmonary Arterial Hypertension

**DOI:** 10.3389/fcvm.2021.719058

**Published:** 2021-09-10

**Authors:** Yanan Sun, Shreya Sangam, Qiang Guo, Jian Wang, Haiyang Tang, Stephen M. Black, Ankit A. Desai

**Affiliations:** ^1^College of Veterinary Medicine, Northwest A&F University, Xianyang, China; ^2^State Key Laboratory of Respiratory Disease, National Clinical Research Center for Respiratory Disease, Guangdong Key Laboratory of Vascular Disease, Guangzhou Institute of Respiratory Health, The First Affiliated Hospital of Guangzhou Medical University, Guangzhou, China; ^3^Department of Medicine, Krannert Institute of Cardiology, Indiana University, Indianapolis, IN, United States; ^4^Department of Critical Care Medicine, Suzhou Dushu Lake Hospital, The First Affiliated Hospital of Soochow University, Suzhou, China; ^5^Department of Cellular Biology and Pharmacology, Herbert Wertheim College of Medicine, Miami, FL, United States; ^6^Center for Translational Science and Department of Environmental Health Sciences, Robert Stempel College of Public Health and Social Work, Florida International University, Port St. Lucie, FL, United States

**Keywords:** pulmonary arterial hypertension, estrogen paradox, estrogen metabolism, estrogen receptors, pulmonary hypertension

## Abstract

Pulmonary arterial hypertension (PAH) is a complex and devastating disease with a poor long-term prognosis. While women are at increased risk for developing PAH, they exhibit superior right heart function and higher survival rates than men. Susceptibility to disease risk in PAH has been attributed, in part, to estrogen signaling. In contrast to potential pathological influences of estrogen in patients, studies of animal models reveal estrogen demonstrates protective effects in PAH. Consistent with this latter observation, an ovariectomy in female rats appears to aggravate the condition. This discrepancy between observations from patients and animal models is often called the “estrogen paradox.” Further, the tissue-specific interactions between estrogen, its metabolites and receptors in PAH and right heart function remain complex; nonetheless, these relationships are essential to characterize to better understand PAH pathophysiology and to potentially develop novel therapeutic and curative targets. In this review, we explore estrogen-mediated mechanisms that may further explain this paradox by summarizing published literature related to: (1) the synthesis and catabolism of estrogen; (2) activity and functions of the various estrogen receptors; (3) the multiple modalities of estrogen signaling in cells; and (4) the role of estrogen and its diverse metabolites on the susceptibility to, and progression of, PAH as well as their impact on right heart function.

## Introduction

Pulmonary arterial hypertension (PAH) is a complex and devastating disease characterized by a progressive increase in pulmonary vascular resistance (PVR) and subsequent, right heart failure, considered the most common cause of death in patients (1). The updated hemodynamic criteria define pulmonary hypertension (PH) using a threshold for mean pulmonary artery pressure (mPAP) > 20 mm Hg, pulmonary artery wedge pressure (PAWP) ≤ 15 mm Hg, and PVR ≥ 3 Wood units (WU) (2) during a resting right heart catheterization (RHC). Obliterative pulmonary arterial (PA) remodeling, sustained vasoconstriction, reduced wall elasticity, *in-situ* thrombosis in small pulmonary arterioles, and formation of plexiform lesions (PXL) (3) are hallmarks of PAH pathology and contribute to the rise in PVR.

Evidence of sexual dimorphism is a distinguishing feature of developing PAH. The World Health Organization describes sex as a biologically determined characteristic, whereas gender is used to describe a characteristic that is socially constructed (4). Therefore, the term sex is used in this review to discriminate differences between male and female subjects. Furthermore, while the proportion of women with PAH is greater, their survival rate is higher than that of men (5–10). Based on these clinical observations, a significant amount of research has been carried out to investigate the potential roles of estrogen signaling in PAH. Specifically, the identification of protective or pathogenic roles of estrogen signaling provide a complex molecular model to decipher the contrasting clinical observations in both sexes. In some circumstances, published research on estrogen signaling from conventional animal models of PAH appear to directly contradict observations from human disease, often termed the “estrogen paradox.” Female rodents, for example, are less susceptible than male rodents to the development of PAH (11, 12). Indeed, these observations along with other findings provide more credence to the notion that established animal models do not fully mimic PAH in patients (11). Indeed, these contradictory findings from animal models emphasize the need for caution in interpreting the translatability as well as the importance of choosing the appropriate animal models when studying the sexual dimorphism of PAH. This review will summarize our most up to date knowledge regarding the sex differences in existing pre-clinical models of PAH, including data from novel transgenic models (13–15), that provide novel insights into the sex differences related to estrogen signaling in PAH. In the process, we will highlight the importance of selecting suitable animal models for PAH research.

Estrogen signaling is complex and requires the evaluation of both the hormone itself and its metabolites. As a sex steroid hormone, estrogen is produced through multiple reactions and has intricate catabolic pathways *in vivo*. Some of its metabolites have been reported to promote the development of PAH, while others have been shown to attenuate PAH development. A careful inventory of each metabolite and its function is critical to better understanding the estrogen paradox. Moreover, both PAH status and progression, themselves affect the synthesis and catabolism of estrogen. This latter observation is based on prior studies (16–18) that reported changes in estrogen-metabolizing enzymes and the proportion of estrogen metabolites during PAH development. The relationship between estrogen and PAH, therefore, appears bidirectional in terms of cause and effect. Further, beyond circulating levels, local concentrations of estrogen signaling mediators also appear to be important in PAH. For example, estrogen-metabolizing enzymes are tissue-specific, raising questions about possible discrepancies between the levels of estrogen (and its metabolites) in the circulation vs. in the heart and lungs. Whether the presence of PAH or its progression leads to changes in the local metabolism of estrogen in the heart and lungs also deserves more attention. In addition, the relationship between estrogen and gene expression is complex. Signal transduction of estrogen is influenced by various factors including, age, sex, receptor type, cell type, disease status, etc. Indeed, the three known estrogen receptors (ERs-ERα, ERβ, and GPER) exhibit different roles in regulating gene expression. Moreover, dimers formed by ERα and ERβ may manifest as hetero- or homodimers and function *via* individual or dimer forms, further complicating estrogen signaling (19). This review will further discuss estrogen signaling in the context of the relative content of ERs, ERs' different position (membrane or nucleus) in cells, and varying modifications of ERs, to give a full picture of the sex-specific differences and the need for the development of sex-specific treatments in PAH.

In order to better understand the estrogen paradox, this review will summarize available evidence and discuss the following: (1) potential origins of sex differences; (2) mechanisms involving estrogen anabolism and catabolism; (3) the diverse cellular roles of ERs; (4) multiple modes of estrogen signaling; (5) direct and indirect effects of estrogen hormone and its metabolites. These discussions will also shed insight into the impact of both the presence and severity of PAH on estrogen anabolism and catabolism, ERs, and estrogen metabolites.

## Sex Differences in PAH

### Sex Differences in Patients With PAH

As early as the 1950s, a predilection was observed for female patients in PAH (20, 21), a finding that has since been confirmed by multiple studies (7–9, 22). In 1987, National Institutes of Health (NIH) registry reported the ratio of male to female patients in primary PH was 1–1.7. This data, however, did not break down PH into the current sub-group classifications (5). The findings of the French National Registry of PH also suggested that women account for a greater proportion of patients with most PAH subtypes, such as idiopathic, familial, anorexia, connective tissue disease and congenital heart diseases associated PAH (6). In hereditary PAH, the incidence of PAH among carriers of bone morphogenic protein receptor type II (*BMPR*2) mutations also varies by sex−42% for women and 14% for men (7). Sexual dimorphism is also reflected in surveys across geographical regions and nationalities. Data from the Japan PH Registry (JAPHR) showed that 76.2% of Japanese patients with PAH were female (8). The Registry to EValuate Early And Long-term pulmonary arterial hypertension disease management (REVEAL Registry) shows a greater number of women with the disease in the US (9, 22). These and other registries, composed of mostly adult patients, consistently show a female predilection (Table 1). Differences in exact rates across these registries may reflect different inclusion/exclusion criteria, drug exposure history, lifestyle differences, among other confounders. Notably, hemodynamic criteria for PAH in these registries were based on older definitions: mPAP ≥ 25 mm Hg at rest; these data did not include any patients whose mPAP was between 20 and 24 mm Hg based on the new definition of PAH with a lower threshold of mPAP (≥20 mm Hg). Interestingly, Hoeper et al. re-analyzed the results of the Comparative Prospective Registry of Newly Initiated Therapies for Pulmonary Hypertension (COMPERA) registry and stratified patients into two categories: old (>65 years old) and young (18–65 years old), they reported a sex difference was apparent in young but not older subjects (25).

**Table 1:** Proportion of female patients in major PAH registries.

**Registry**	**Median age, yrs**		**Female, %**		**Study design and time period**	**No. of patients (female)**	**References**
	**PAH**	**IPAH**	**PAH**	**IPAH**			
USA NIH	–	36	–	63%	1981–1985	187	(5, 23)
China	–	36	–	71%	1999–2004	72 (51)	(23)
UK/Ireland	–	50	–	70%	2001–2009	482 (337)	(24)
COMPERA	–	65	–	60%	2007–2011	587	(25)
USA PHC	48	–	77%	–	1982–2006	578	(26)
Czech Republic	52	–	65%	–	2000–2010	191	(27)
USA REVEAL	50	–	80%	–	2006–2007	2,525	(22)
COMPERA	68	–	64%	–	2007–2013	1,283 (819)	(28)
JAPHR	44	–	76%	–	2008–2013	189 (144)	(8)
Scotland SMR	51	49	70%	62%	1986–2001	374 (261)	(29)
Mayo	52	52	75%	76%	1995–2004	484	(30)
Spanish	45	46	71%	73%	1998–2006; 2007–2008	866	(31)
France	50	52	65%	62%	2002–2003	674	(6, 32)

While female patients are more susceptible, mortality data consistently show that they exhibit better survival outcomes than males. For example, in the Spanish Registry of Pulmonary Arterial Hypertension, male sex is an independent predictor of death, associated with an increased risk of death (31). The REVEAL registry also reported a worse prognosis for men than women (33) and the French registry showed female sex was an independent predictor of survival (34). Data from the COMPERA registry showed similar survival benefits for female patients (28). These observations cumulatively establish significant differences between male and female patients with PAH including disease risk and outcomes.

### Sex Differences in Animal Models of PH

To better understand sex differences in patients with PAH, several animal models have been studied. Interestingly, while sex differences are also found in animal models of PH, not all are consistent with those observed in patient studies. We summarize sex differences in four conventional animal models of PH: Sugen/Hypoxia induced rodent PH (SuHx-PH), monocrotaline-induced rodent PH (MCT-PH), chronic hypoxia-induced PH (HPH), and genetically modified animal models of PH.

Female rats manifest a milder form of disease severity than male rats when exposed to HPH or MCT-PH. Ovariectomy (OVX) further exacerbates PH in female rats, and exposure to OVX-treated animals with 17β-estradiol (E2) alleviates PH in these animal models (11, 12, 35). Apart from improved right heart function and survival advantages, female mice have less PA remodeling than male mice in HPH (13). Moreover, vascular occlusion and PXL are not observed in the HPH model (11). These findings appear to be inconsistent with the suspected pathogenic role of estrogen in pulmonary blood vessels of patients. It is also worth noting that the MCT is biologically activated in the liver by cytochrome P-450 (CYP-450) into MCT pyrrole (MCTP) (36), which can damage the lung endothelium and leads to the development of PH. Studies have shown a sex-specific role for CYP-450 involvement in the metabolism of MCT in the liver (36), which may partly explain sex discrepancies between animal MCT-PH models and patients.

In the SuHx-PH model, although female rats exhibit more pronounced smooth muscle thickening of the pulmonary vasculature, they display better cardiac function and a better ability to cope with increased afterload caused by acute exercise challenge than male rats (37). The survival rate of female rats is also higher than in males. OVX treatment exacerbates the severity of SuHx-PH in female rats, and supplementing with exogenous E2 after OVX, improves right heart function and PA remodeling. The increased severity in vascular remodeling, improved right ventricular (RV) adaptability, and superior survival rate of female rats in the SuHx-PH model better mimic those observed in PAH patients. Comparing these features from HPH and MCT-PH models, SuHx-PH may represent a more suitable model to study sex differences in PAH.

Select transgenic models have also successfully imitated the increased susceptibility in women observed in patients with PAH and may be leveraged to investigate the role of a targeted pathway in PAH-related sexual dimorphism. For example, in signal transducers and activators of transcription 5 heterozygous (*Stat5*^+/−^) or homozygous (*Stat5*^−/−^) mice, female mice exhibited more severe PH than males after exposure to hypoxia (13). In addition, in mice over-expressing the calcium-binding protein S100A4/Mts1, right ventricle systolic pressure (RVSP) and pulmonary vascular remodeling were increased in female mice, while male mice were unaffected (14). In mice over-expressing serotonin transporter (SERT^+^), female mice showed increased RVSP and PVR, while male mice were unaffected. Moreover, OVX eliminates the effect of SERT^+^ in females. Also, long-term administration of E2 re-established the PAH phenotype after OVX in both normoxic and hypoxic SERT^+^ mice (15).

### The Estrogen Paradox in PAH—Lessons From Animal Models

Although PAH is considered a disease of the pulmonary vasculature, the ability of the right heart to adapt to the increased PVR determines the survival rate in patients with PAH (32–34, 38). Registries of PAH show that female patients with PAH have more favorable hemodynamic characteristics and better RV function than male patients (39, 40), including higher cardiac index (CI) and lower RVSP, lower average PA pressure and PVR (41, 42). Subsequent animal studies have also shown that estrogen mainly exhibits protective effects on the RV, while in pulmonary blood vessels, its role is relatively complex. In the pulmonary vasculature of animal models, the pathogenic effect of estrogen may be greater than the protective effect, but these findings remain unclear. Nonetheless, these animal model observations may partly explain why women are more susceptible to disease risk but have a lower mortality rate in PAH.

## Estrogen Synthesis and Metabolism

To dissect the underpinnings of the sexual dimorphism and the estrogen paradox, significant attention has been paid to estrogen synthesis, signaling, and metabolism and their contribution to the development of PAH. Given the diversity of estrogen, its metabolites, as well as some estrogen analogs, it is important to discuss evidence of their involvement and potential as therapeutic targets in PAH.

**Figure 1:**
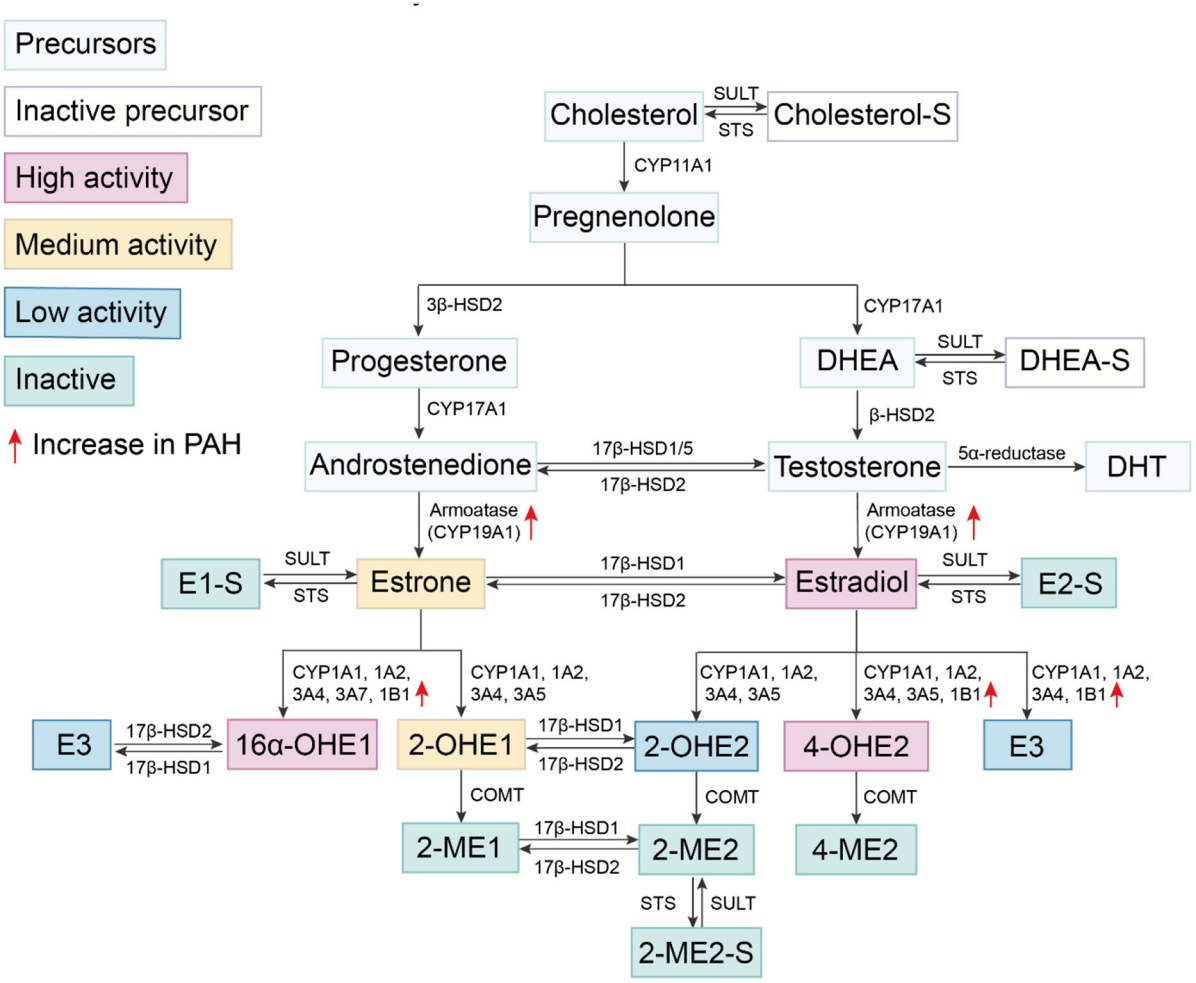
Estrogen synthesis and metabolism. Cholesterol is catalyzed by the cytochrome P450 enzyme and undergoes several conversions to become DHEA. DHEA is converted to testosterone under the catalysis of different β-HSDs. Androstenedione and testosterone are converted into estrone and E2 by CYP19A1, respectively. E2 is oxidized on carbon at multiple positions, including oxidation at the C17 position by 17β-HSD responsible for the reversible conversion between E1 and E2. Other oxidation sites include those at C2, C4, and C16 positions and produce different metabolites *via* varying cytochrome P450 enzyme. E1, E2, estrogen precursors and some metabolites can be combined with sulfonate groups by SULT to convert them into sulfuric steroids. Sulfation of steroids can be converted into active forms by STS.

The basic metabolic pathways of estrogen synthesis are important to briefly summarize when considering their role in PAH. The initial step in the synthesis of sex hormones (Figure 1) is the conversion of cholesterol to pregnenolone under the action of cytochrome P450 (CYP) enzymes (43). Pregnenolone is converted to progesterone by 3β-hydroxysteroid dehydrogenase (3β-HSD). Pregnenolone and progesterone are oxidized to dehydroepiandrosterone (DHEA) and androstenedione under the action of CYP17A1, respectively. They are transformed into testosterone under the action of different β-hydroxysteroid dehydrogenase 1 (β-HSD1). The final step in the production of estrogen is the conversion of androstenedione or testosterone into estrone (E1) or E2 under the action of aromatase (CYP19A1), respectively. Notably, aromatase is increased in the lungs of female patients with PAH (16), suggesting that PAH development is associated with increased estrogen production. Estrogens produced by the human body include E1, E2, and estriol (E3). Among them, E2 is the most abundant estrogen with the strongest biological activity, and it is one of the main female hormones produced by the ovaries. E1 and E3 are weak estrogens. E1 is increased in post-menopausal females. E3 is the degradation product of E2 and is increased during pregnancy.

E2 is oxidized at multiple carbon positions. Oxidation at the C17 position is required for the conversion between two estrogens, E1 and E2 by 17β-HSD; specifically, E1 converts to E2 *via* 17β-HSD1 while E2 converts back to E1 *via* 17β-HSD2. This process is reversible, but the rate of conversion from E1 to E2 is slower. Other sites of oxidation of E2 predominantly occur at C2, C4, and C16 positions which results in the production of different metabolites. These estrogen metabolites have previously been shown to exert diverse biological effects in PAH (44–46).

### E2 Catabolism at Position C2

Hydroxylation at the C2 position represents one of several available pathways for E2 metabolism (47). The metabolites produced from this pathway have minimal to no estrogen activity, thereby, rapidly reducing the amount of active estrogen. CYP450 isomers CYP1A1, 1A2, 3A4, 3A5, etc. catalyze conversion of E2 to form 2-hydroxyestradiol (2-OHE2) (47). 2-OHE2 has low estrogen activity and is further converted into 2-methoxyestradiol (2-ME2) *via* catechol-O-methyltransferase (COMT). 2-ME2 has no detectable estrogenic activity (48, 49). Hydroxylation of E1 at this C2 position generates 2-hydroxyestrone (2-OHE1), which has a higher ability to bind ERα than ERβ (50). Conversion of both E1 and E2 into other metabolites predominantly occurs in the liver. Cardiovascular tissues also facilitate conversion of E2 into 2-OHE2 and 2-ME2, such as cardiac fibroblasts (51), vascular endothelial cells (EC) (52), and smooth muscle cells (SMC) (53).

### E2 Catabolism at Position C4

While hydroxylation at the C4 position accounts for only a small portion of E2 hydroxylation, the resulting metabolite, 4-hydroxyestradiol (4-OHE2), has high estrogenic activity. These C4 reactions are catalyzed by enzymes such as CYP1A1, 1A2, 3A4, 3A5, and 1B1. The latter, CYP1B1, catalyzes the reaction of C4 for the most part, while the other enzymes primarily catalyze the reaction at C2, followed by the reaction at C4 (47). Notably, 4-OHE2 reportedly exhibits reduced binding affinity with ERs, is reactive oxygen species (ROS)-dependent and has carcinogenic effects (54). Furthermore, similar to the metabolic reaction at the C2 position, 4-OHE2 is converted into 4-methoxyestradiol (4-ME) *via* catechol-O-methyl transferase (COMT) or into 4-hydroxyestrogen (4-OHE1) *via* 17β-HSD.

### E2 Catabolism at Position C16

Hydroxylation of E2 at the C16 position is also one of the main mechanisms for the formation of highly estrogenic metabolites. The enzymes involved include CYP1A1, 1A2, 3A4, 3A7, and 1B1 (55). Among them, CYP3A7 has high catalytic activity for 16α-hydroxylation of E1, but not for E2 (56). E1 and E2 are metabolized to 16α-hydroxyestrone (16α-OHE1) and 16α-hydroxyestradiol (E3, estriol), respectively (47). E3 is only produced in large amounts during pregnancy. Although the estrogenic activity of E3 is weak, it can be catalyzed by 17β-HSD2 to produce 16α-OHE1. 16α-OHE1 is a highly estrogenic metabolite, more potent than the highly abundant E2, and has a slightly higher affinity for ERβ than for ERα (50). Moreover, 16α-OHE1 also prolongs ER activation (57, 58). In patients and various experimental PAH models, the expression of CYP1B1 is also increased, especially in the pulmonary vasculature (17, 46, 59). CYP1B1 catalyzes the catabolism of estrone to 16α-OHE1 in extrahepatic tissues. Austin et al. compared the levels of estrogen metabolites in female subjects with *BMPR2* mutations, and found that 2-OHE: 16α-OHE1 ratio of affected mutation carriers was 2.3 times lower than that of unaffected mutation carriers (18). The accumulation of 16α-OHE1 and 16α-OHE2 has been independently validated in idiopathic PAH patients (60). Broadly, these studies indicate dysregulation of estrogen catabolism in PAH. Dexfenfluramine (Dfen)-induced PH (59), HPH, and SuHx-induced PH (46) are all attenuated in *CYP1B1* knockout (KO) mice. The selective CYP1B1 inhibitor, 2,3′,4,5′-tetramethoxystilbene (TMS), attenuates Dfen-induced PH, primarily by inhibiting estrogen-induced proliferation of human PASMCs (46). Therefore, PAH development may predispose estrogen catabolism toward 16α-OHE1 generation. By increasing 16α-OHE1, a series of PAH-promoting estrogen effects are potentially amplified. However, this hypothesis requires further validation.

### Enzymes Involved in Estrogen Synthesis and Metabolism

Estrogen metabolism and catabolism are well-known but its characteristics across tissue-types remain poorly studied. In PAH, emerging data indicate parallel complexity and specificity based on disease-relevant tissues including EC, SMC, and cardiomyocytes. There are two obvious changes in estrogen metabolism in PAH. Aromatase is increased in the lungs of female patients (16) while CYP1B1 is increased in patients and various experimental PAH models, which in turn increases the 16α-OHE (17, 46, 59, 60). The enzymes responsible for estradiol synthesis are expressed in the liver as well as in vascular SMCs (61), cardiac fibroblasts and cardiomyocytes (62). Moreover, the CYP subtypes involved in the oxidative metabolism of estrogen have different levels of catalytic activity and unique regioselectivity (47). For example, estrogen catabolism in liver tissue is mainly mediated by CYP1A2 and 3A4, while in extrahepatic tissue, it is catalyzed by CYP1B1 and 1A1 (55). CYP1B1, 1A1, as well as COMT were all observed in cardiovascular tissues (51, 62–64). These finding demonstrate that estrogen can be locally synthesized and metabolized in the blood vessels of heart and lungs, tissues where the effects of estrogen and its metabolites likely target during PAH pathogenesis.

### Sulfation Pathway

Combining sulfonate groups, sulfotransferase (SULT) converts E1, E2, estrogen precursors and metabolites (DHEA, 2-ME, etc.) into sulfuric acid E1, sulfuric acid E2, sulfuric acid 2-ME, etc. These sulfated steroids are considered inactive, cannot activate ERs, and also hydrophilic, which makes them easier to excrete through the kidney (65). In various cancer cell lines, increased SULT activity is related to the weakening of the anti-mitotic effect of 2-ME2 (66). Specifically, the level of activity of these steroids are reduced by sulfation. Sulfation of steroids, however, can further be converted into active forms by sulfatase (STS). Therefore, SULT and STS regulate the content of biologically active estrogen, its precursors and metabolites depending on whether they are sulfated.

## Estrogen Receptors

Estrogen exerts most of its biological functions through ERs. ERs are composed of a DNA-binding domain, a ligand binding domain, and an amino terminal transcription control domain (AF-1). ERs interact with regulatory binding proteins through AF-1, a region with significant differences between ERα and ERβ (67). GPER, a 7-pass transmembrane G protein-coupled ER that is predominantly localized in the endoplasmic reticulum (68), binds estrogen with high affinity to activate several cellular signaling cascades (69). GPER is an orphan receptor unrelated to nuclear ERs (nERs), which mimics the binding and signaling characteristics of membrane ERs (mERs) (70). There are three receptors expressed in lung (71–74) and heart tissue (75, 76). While both, ERα and ERβ, are expressed in PAECs (77) and PASMCs (78), ERβ is primarily expressed in PAECs (79). GPER is seen in the intima and media of the aorta by immunohistochemical staining (75), and its abundance is similar in males and females. Studies on the roles and expression of ERs in the pulmonary vasculature have not been as exhaustive as those in the systemic vasculature, and much of our knowledge is still derived from animal studies.

### Regulation of ERs Activity

The content and ratio of ERα and ERβ influence estrogen effects on gene expression. ER levels appear to be different in men than in women, and in women, before and after menopause. Gavin et al. described that early follicular ERα expression was 30% lower than that of late follicles in vascular ECs of premenopausal women, and ERα levels were 33% lower than that of late follicles in postmenopausal women (80). In addition, although Grohe et al. did not observe any sex differences in the expression of ERβ in cardiomyocytes, a significant change in ERα expression by sex was observed (71). GPER is also affected by sex and age. The expression of GPER in the mesenteric artery of men and elderly women is reduced by ~50% compared with young women (81). The abundance of ERα and ERβ in different tissues and cells may vary (82). In the lung, ERβ is reportedly more abundant than ERα (83, 84). The relative function of a specific ER may further depend on the clinical context, for example, men or women, premenopausal women or postmenopausal women.

In addition to age and sex, disease factors also influence ER expression levels in the cardiovascular system. Cardiac pressure load, for example, increases both, ERα and ERβ, in the human heart (85), while heart failure increases ERα (86). ERα expression is reduced in the RV of SuHx-PH when they are subject to OVX and this recovers after E2 supplementation (87). The expression of ERα in the RV is negatively correlated with RVSP and right ventricular hypertrophy (RVH) (87). Similarly, Pinzone et al. found that estrogen positively regulates ER levels (88). Ihionkhan et al. reported that chronic use of exogenous estrogen modifies transcription of ERα gene in the endothelium through ERα or ERβ, such that the expression of ERα is increased while ERβ is decreased (89). Further, increased expression of ERα in female PAH patients correlates in increased PASMC proliferation and remodeling (79) *via* multiple pathways including MAPK and Akt signaling. In contrast, in hypoxic male rats, increased ERβ, but not ERα, expression is observed in pulmonary vessels (90). Also, the expression of ERβ mRNA in male blood vessels is increased after vessel injury (91). Moreover, the biological functions of ERβ may depend on the presence of ERα in certain cell types and tissues (92). Therefore, when discussing the role of ERs activity in PAH, it is necessary to pay attention to the effect of PAH on ERs in the heart and lungs.

Modification events also influences ER activity. For example, the methylation of the ER promoter reduces ER expression (93) while acetylation increases the transcriptional activity of ERα (94) and enhances the activity of ERα-dependent gene regulation (95). In addition, the phosphorylation of ER exerts ER ligand-independent regulation, enhancing ER signaling (96). NO-mediated S-nitrosylation of ER has also been shown to occur at the two major DNA-binding zinc-finger domains of ER and this leads attenuated binding to specific estrogen response elements (EREs) and may potentially favor activation of non-genomic signaling pathways (97).

### Differences Across ERs and Their Roles in Regulating Gene Expression

As mentioned above, ERs perform different functions according to both the recruited co-regulators and the bound transcription factors (TFs). Studies have also shown that ERα and ERβ may have opposing effects in the same tissue (98). For example, in vascular SMCs, ERβ up-regulates the expression of inducible nitric oxide synthase (iNOS) while ERα causes down-regulation (99). In addition, ERα has been shown to mostly up-regulate gene expression in the mouse aorta while 90% of estrogen-mediated genes were downregulated in an ERβ-dependent manner (82). Conversely, in the hearts of female mice, 122 genes were up-regulated and only 23 genes were down-regulated by ERβ. The gene ontology “muscle contraction” was down-regulated after using an ERβ-selective agonist, diarylpropionitrile (DNP), whereas ERβ upregulated immune/chemokine genes and genes involved in regulating cell death (100). Together these findings demonstrate that ERα and ERβ have divergent effects, not only in their abundance and functions, but also in the types of genes they regulate downstream as well as across different tissues. Importantly, the effect of ERs on gene expression may also be time-dependent (101). Schnoes et al. reported that estrogen recruits specific TFs in vascular tissues in a fast and temporary manner (102). Recent studies showed that the method of administering estrogen or ER agonists can be divided into acute (ranging from several minutes to several hours) or chronic (ranging from 1 week to several weeks), which may work through different estrogen signaling pathways.

The complexity of ER-mediated gene regulation may represent a significant reason underlying the “estrogen paradox” in PAH. The lack of comparative studies on the distribution and proportion of ER subtypes in and between humans and animal models further compound the assumptions in translation. Specifically, they raise the question of reliability of using animal models to mirror estrogen signaling in humans. In addition, the signal transduction of ERs requires the participation of co-regulators. The type, level and post-translational modification of co-regulators will affect the signal transduction of ERs. Research on these aspects remains scarce, and subsequent studies will need to define the impact of these factors on PAH development.

### Differences in the Roles of ERs in Disease

ERα and ERβ show contrasting effects during disease states through the regulation of different genes. Overall, ERβ appears to exhibit a protective role in cardiopulmonary diseases, and has shown to exert anti-fibrotic (103), anti-hypertrophic (104), anti-inflammatory (12) and vaso-dilatory properties (105). ERβ has been shown to promote the production of various angiogenic factors to regulate angiogenesis, and NO, exerting vasodilator properties, mediating protective effects of estrogen in PAH. ERβ agonists have also been used to treat PAH (12). Further, studies support an important role for ERα during E2 signaling in PAH. These include data demonstrating that ERα mainly mediates the effect of E2 on cardiopulmonary hemodynamic parameters in HPH (90), that there are minimal to no effects of estrogen on vascular injury in *ER*α KO mice that underwent OVX (106) and that ERα in ECs plays a key role in E2-mediated vascular endothelialization (107). GPER has been shown to mediate cardioprotection through the activation of the PI3K/Akt pathway (75). GPER agonist, G1, inhibits the proliferation of cardiac fibroblasts in a dose-dependent manner (76), and alleviates cardiac remodeling and diastolic dysfunction in rats (108). G1 also improves the function and reduces myocardial inflammation during ischemia/reperfusion (109). The stimulation of GPER prevents changes in intracellular calcium concentration and vascular tone caused by vasoconstrictor and inhibits the proliferation of human vascular SMC (74). Conversely, the deletion of the *GPER* gene in mice eliminates the vascular effects of GPER activation (74). Based on these differential responses, selective activation of specific ER subtypes may help elucidate the effects of estrogen signaling on cardiopulmonary function in PAH.

## Estrogen Signaling Pathways in PAH

The role of estrogen signaling in regulating gene expression is well-characterized (110). Multiple modes of signaling have been previously described (19, 96, 110). Two of these pathways are considered “genomic signaling” due to the direct involvement of DNA binding (19, 96), While a third type of non-genomic signaling cascade also has been described, typically *via* protein kinases (110).

### Genomic Signaling Pathway

**Figure 2:**
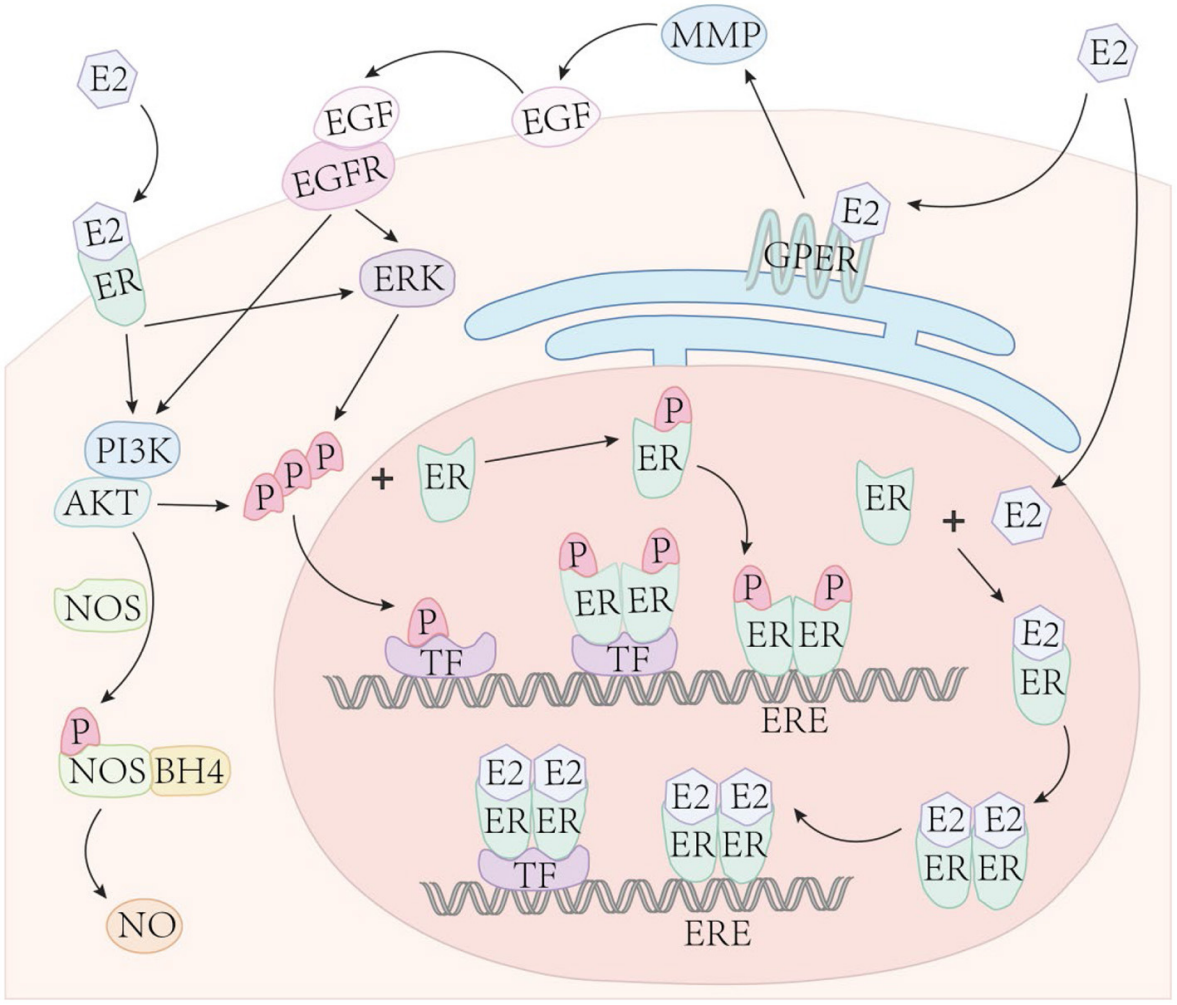
Estrogen signaling. Estrogen enters the cell nucleus and binds with nuclear ERs to form an estrogen-ER complex. The combination of the two estrogen-ER complexes typically lead to dimerization, then bind to DNA in two ways: (1) The estrogen-ER complex directly binds to the ERE sites on DNA and acts as a transcription factor, up- or down-regulating gene expression based on the types of recruited co-regulators; (2) In genes with absent EREs, ER indirectly binds to DNA through other TFs to influence gene expression. Phosphorylated ERs directly binds to ERE or indirectly binds to DNA through TFs, similar to estrogen-ER complexes. Estrogen binds to membrane ERα, Erβ, and GPER to mediate non-genomic (acute) estrogen signaling through the activation of various protein kinase cascades.

Binding of estrogen (and its metabolites) to ERs can directly regulate gene expression and is often referred to as the classical pathway. Specifically, this pathway is initiated when estrogen enters the cytoplasm and binds with nERs to form an estrogen-ER complex (Figure 2). The combination of estrogen-ER complex leads to dimerization [either homodimer or heterodimer (19)] leading to DNA binding in two ways. First, the estrogen-ER complex can directly bind to EREs and acts as a TF complex, resulting in either up- or down-regulation of gene expression according to the types of recruited co-regulators (co-activators or co-suppressors). Second, on genes without EREs, the estrogen-ER complex indirectly binds to DNA through other TFs to regulate gene expression. This specific mechanism is often referred to as transcriptional crosstalk. For example, estrogen interacts with other promoter binding proteins to activate or inhibit AP-1 dependent transcription (111). Notably, many estrogen-responsive genes that lack full ERE sequences contain either partial ERE sequences or the binding site of SFRE, a response element with orphan nuclear hormone receptor (SF-1) that serves as a direct ER binding site (112). To our knowledge, only ERα has been observed to bind to SFRE (113). Importantly, about one-third of human genes are estimated to be targets for ER binding indirectly through intermediate TFs (112).

ERs can also independently interact with DNA without binding to estrogen and regulate gene expression, called ligand-independent transcriptional regulation. This mechanism requires ERs to be phosphorylated at specific serine sites (96). The phosphorylated ER directly combines with EREs or indirectly binds to DNA through TFs, similar to estrogen-ER complexes. This mode of ER ligand-independent transcriptional regulation also requires phosphorylation of growth factor-dependent coactivators. For example, Carascossa et al. demonstrated that although protein kinase A (PKA) is not required to participate in the direct phosphorylation of ERα, PKA assists in the phosphorylation of activator-related arginine methyltransferase 1 (CARM1), which is necessary for ERα ligand-independent transcriptional regulation (114).

### Non-genomic Signaling Pathway

The non-genomic pathway is also a common form of signal transduction of steroid hormones, generally through the activation of various protein kinase cascades (110). Both, ERα and ERβ, present on the cell membrane (115), as well as the estrogen receptor, GPER (also known as GPR30) (116), can mediate non-genomic estrogen signaling. E2 binds to the membrane-bound receptor and leads to the activation of G-protein directly coupled to the receptor, indicating that GPER is a G protein-coupled receptor. The activity of adenylate cyclase is also increased in this mode of signaling (70). Estrogen binds to the above receptors and then activates kinases or secondary messengers, including phosphoinositide 3-kinase (PI3K) (115), mitogen-activated protein kinase (MAPK) (117), etc.

Based on prior work, cardiovascular protection derived from estrogen is mediated, in part, by the activation of non-genomic effects (74, 118, 119). ERα and ERβ both have been shown to induce Akt-dependent activation of endothelial NOS (eNOS) in vascular ECs through a non-genomic mechanism, thereby, rapidly causing vasodilation (115, 118, 120–124). ERα also mediates the activation of eNOS through a MAPK-dependent mechanism (121). MAPK activation plays an important role in mediating the above non-genomic ER effects. The phosphorylation of ERα and ERβ is also mediated by the MAPK signaling pathway. Therefore, the non-genomic pathway can enhance genomic pathways by phosphorylation of ER. GPER activation also shows cardioprotection in both male and female rats (75), The GPER selective agonist, G1, effectively reverses PH-induced RV dysfunction, structural abnormalities and exercise intolerance in male rats (125).

Non-genomic pathways of estrogen conduction may indirectly affect gene expression by activating other signal transduction pathways that can act on target TFs. While the genomic effect takes several hours to manifest, non-genomic pathway is typically considered “acute” and characterized to manifest in a few seconds to minutes.

## Effect of Estrogen and Its Metabolites on PAH

### Cardioprotection

In PAH, mortality is closely related to sex and RV hemodynamic function (32, 39). Even though medical therapy reduces PVR, the prognosis of PAH patients remains poor from RV failure (38). RV ejection fraction (RVEF), assessed by magnetic resonance imaging, is an important determinant of the prognosis of patients with PAH. Increased RVEF indicates a higher survival rate (126). Male patients with PAH display lower RVEF than those of female patients (40). The response of males to heart pressure overload, cardiac contractility, and heart adaptability are also not as strong as females. Among females treated with estrogen, higher E2 levels were also associated with better RV contractile function (41, 42, 127), suggesting that estrogen may have cardioprotective effects.

**Figure 3:**
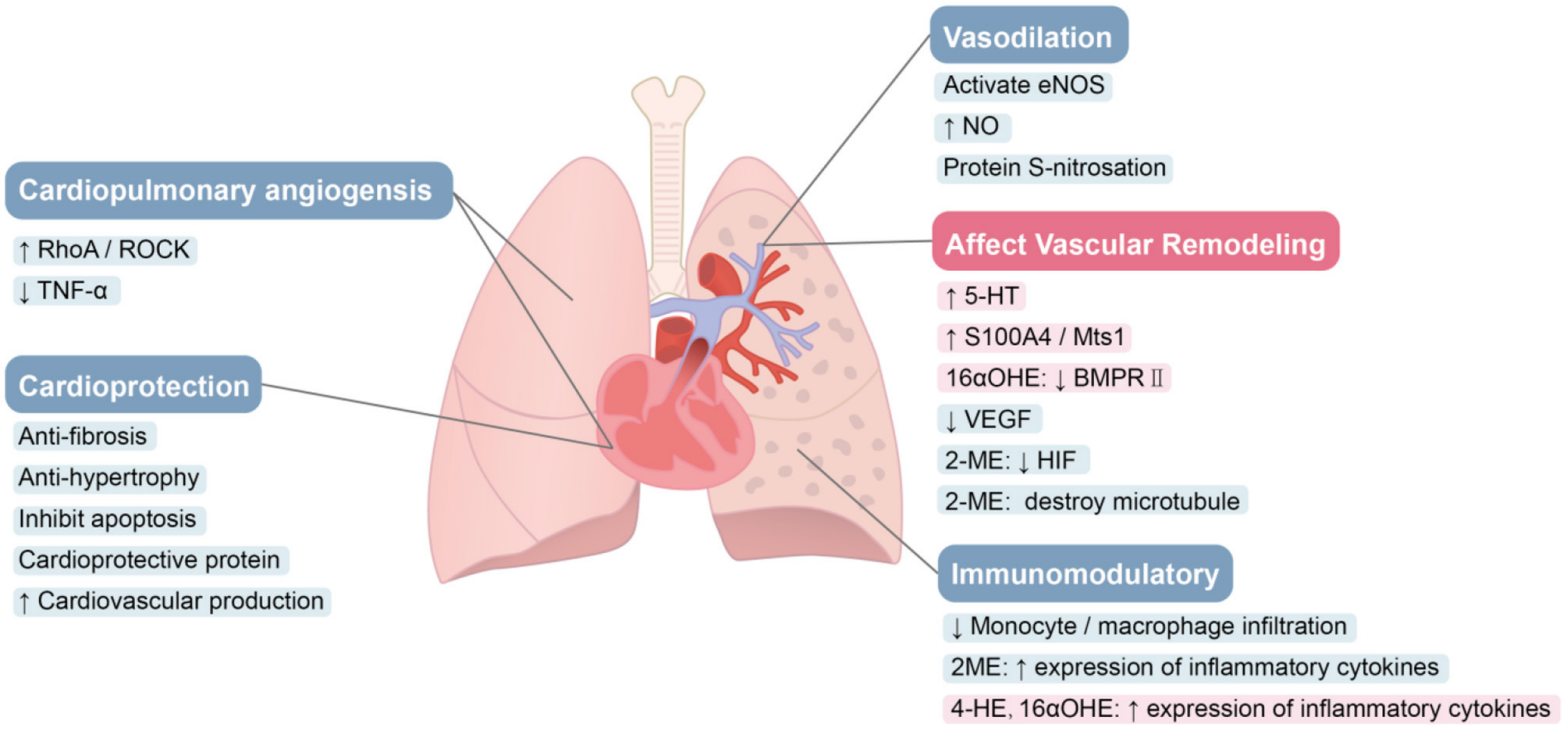
Role of estrogen and its metabolites in PAH. E2 protects the heart through various pathways. Estrogen can promote blood vessel remodeling. E2 improves cardiac structure and function *via* augmentation of angiogenesis. Estrogen can improve pulmonary hemodynamics. Estrogen and 2-ME appear to suppress inflammation in PAH, while 4-OHE and 16α-OHE1 have significant pro-inflammatory effects.

E2 protection in the heart has been reported to signal through various pathways (Figure 3). In one study, the conversion of cardiac fibroblasts to myofibroblasts is inhibited by estrogen through ERβ, thereby preventing myocardial fibrosis (103). The expression of fibrosis markers and metalloproteinases in cardiac fibroblasts were directly inhibited by E2. Reversing fibrosis and up-regulating the expression of new extracellular matrix enzymes such as ADAM15, ADAM17 and OPN was associated with E2's ability to reverse the adverse RV remodeling associated with PH. These effects also may be mediated by ERβ (128). ERβ not only weakens fibrosis, but also inhibits the development of apoptosis, thereby slowing the progression of heart failure, which was more obvious in females (129). In addition, estrogen exhibits an inhibitory effect on the hypertrophic response caused by stress overload through the ERβ genomic effects (104, 130, 131). Estrogen also protects the heart through ERβ-mediated cardioprotective protein S-nitrosation (SNO), which requires the involvement of NOS (132, 133). Umar et al. found that recovery after removing estrogen still improves the structure and function of RV in MCT rats and rescues the original severity of PH. The beneficial effects of estrogen in PH appear to come predominantly from its protection of the heart, including stimulation of cardiopulmonary neovascularization, inhibition of fibrosis, RVH (12) and stimulating RV contractility (134).

The protective effects of estrogen on the heart are considered to be partly mediated by ERβ (12). Many protective genes such as *GADD45*β and *COX-2* are up-regulated by ERβ selective agonist, DPN (100). ERβ-mediated PI3K/Akt signal transduction was shown to improve myocardial function (135). Calcineurin-related hypertrophy is also inhibited by ERβ (130). Furthermore, activating ERβ reduces cardiac hypertrophy and fibrosis caused by AngII signaling (104). This finding was confirmed in *ER*β KO mice (136). However, ER-α-mediated acute myocardial protection in female has also been reported, and differential activation of MAPK may mediate this protection (137). GPER has also shown cardioprotective effects in both male and female rats (75, 109, 138).

### Vascular Remodeling

Vascular remodeling in PAH leads to occlusion of the lumen and a resulting rise in PVR. Abnormal proliferation of PASMCs plays a pivotal role in this remodeling (139). Estrogen and its metabolites have key roles in the pathological process. E2 promotes PA remodeling (Figure 3). In PASMCs, E2 induces increased expression of the calcium binding protein, S100A4/Mts1, and activates its endogenous receptor (receptor for advanced glycosylation end products; RAGE), thereby stimulating cell proliferation (14). Serotonin-induced human PASMC proliferation is mediated by SERT and 5-HT1B receptors (140, 141), and E2 mediates the upregulation of SERT and 5-HT1B receptors (15). Increased SERT expression causes increased 5-hydroxytryptamine (5-HT) in cells, and both E2 and 5-HT increase CYP1B1 expression in PAH-derived PASMCs (59). Thus, the vascular remodeling in PAH may be associated with dysregulation of estrogen metabolic pathways through increased CYP1B1 activity and the formation of pathogenic metabolites that promote PASMC proliferation. E2 also promotes PASMC hyperproliferation by inhibiting BMPR2 expression (142–144). Even in normal subjects, the expression and activity of BMPR2 is sex-specific. In PASMCs, the mRNA and protein expression of BMPR2 in females was observed to be lower than that of males in control subjects, and in mice (142), which may contribute to the proliferation phenotype of female PASMCs, and may partly explain why women are more susceptible to PAH (145). BMPR2 signal transduction is reduced in the lungs of SuHx-PH rats. Inhibition of estrogen synthesis with aromatase inhibitors not only reverses SuHx-PH in female rats, but also restores BMPR2 signaling (16), indicating that endogenous estrogen has an inhibitory effect on BMPR2 signaling. Moreover, there is direct binding of ERα to EREs on the promoter of the *BMPR2* gene, resulting in estrogen-mediated reductions in BMPR2 expression (142). In addition, changes in oxygen concentration can alter E2 effects on BMP signaling in human PAECs (146).

Metabolites of estrogen also play a significant role in the abnormal proliferation of PASMCs. The highly estrogenic metabolite, 16α-OHE1, induces the proliferation of human PASMCs isolated from PAH patients (18, 46). 16α-OHE1 has also been shown to stimulate nicotinamide adenine dinucleotide phosphate oxidase (NOX)-induced ROS generation and cause abnormal proliferation, *via* Erα, in human PASMCs (44). *In vivo*, chronic 16α-OHE1 exposure in *BMPR2* mutant male mice doubles the prevalence of PAH and decreased cardiac output. In the whole lungs of control mice, 16α-OHE1 inhibits BMPR2 protein and BMP signaling, but not in *BMPR2* mutant mice, indicating a role for BMPR2 inhibition in 16α-OHE1-mediated proliferative effects.

2-ME2 has been shown to significantly reduce angiogenesis and remodeling. Using *COMT KO* mice, Lefteris et al. demonstrated the vascular anti-mitotic effects of E2 derived from catabolism to 2-ME2, which was ER-independent (147). SMC mitosis is inhibited by 2-ME2 through inhibition of key cell cycle regulatory proteins, up-regulating COX-2, reducing HIF protein expression, interfering with the polymerization of tubulin and disrupting the microtubule network (45, 148–150). The synthetic analog of 2-ME, 2-ethoxyestradiol (2-EE), can also inhibit vascular remodeling in PH in a dose-dependent manner. Moderate concentrations inhibit the growth of human PASMCs *in vitro*, and have a biphasic effect on the growth of ECs (low stimulation and high concentration inhibitory effect) (151). Furthermore, in primary rat aortic SMCs (152) and rat liver epithelial cell lines (153), the expression of angiotensin type 1 receptor (AT1R) was down-regulated by 2-ME2, a response mediated by GPER. Up-regulation of AT1R expression and signal transduction has been shown to be important in pulmonary vascular remodeling in PAH (154). Notably, E2 protection in HPH does not require conversion of E2 to downstream metabolites such as 2-ME2. E2 also directly reduces the secretion of vascular endothelial growth factor (VEGF) in hypoxic rat PAECs in an ER-dependent manner. E2 can inhibit the cell cycle, and reduce vascular remodeling and hemodynamic parameters in HPH through direct antiproliferative effects (90). As 2-ME2 does not appear to interact with nuclear ERα and ERβ in the cardiovascular system the anti-mitotic effects of 2-ME2 may be ER-independent (64).

The effects of E2 on vascular remodeling may be influenced by several factors. Oxygen concentrations can impact E2 signaling and there is a dichotomous effect of E2 in cultured PAECs, with E2 inhibiting hyperplasia pathways during hypoxia but promoting cell proliferation under normal oxygen levels (90). It should be noted that studies that have observed estrogen-promoting effects on angiogenesis and remodeling have all been conducted during normal oxygen levels. Dose-dependence is also important in E2 signaling (151, 155). Metabolite specificity also drives directionality in estrogen signaling. E2 metabolites exert both, proliferative and anti-proliferative, actions. For example, 2-ME is known to have anti-proliferative effects on cells, while 16α-OHE1 stimulates cell proliferation (156). Tissue specificity can influence estrogen signaling. ERα has pro-proliferative properties in some tissues and cancers, while ERβ has anti-proliferative effects (157). The activity of each ER subtype in tissues may also influence the relationship between estrogen and vascular remodeling.

The diverse array of E2 metabolites and their varying biological activities as well as signaling pathways combined may contribute to the diverging effects of estrogen (158). They may explain some of the opposing actions of estrogen metabolites on angiogenesis and remodeling across different studies. Equally important, disruption in the balance of estrogen metabolites may also explain the differential effects of estrogen in animal models of PH (15).

### Angiogenesis

Loss of pulmonary microvessels and impaired regeneration leads to the progression of PAH. The loss of RV myocardial microvessels causes RV ischemia, which is directly related to RV dysfunction (159). Promoting the regeneration of “lost” distal vasculature is considered to be an important future treatment direction (160). Experimental studies have shown that estrogen plays a role in these phenotypes. For example, in MCT-PH, “loss” or pruning of blood vessels can be reversed by E2 treatment. E2 also stimulates the growth of new capillaries in healthy control rats beyond normal levels (12). Furthermore, E2 enhances EC activity during neovascularization (161), enhances EC motility through ERα (162), and promotes functional endothelial recovery after traumatic de-endothelialization injury (163). Tumor necrosis factor-α (TNF-α)-induced EC apoptosis is also inhibited by E2 in a dose-dependent (155). E2 enhances RhoA/ROCK signaling through ERs and increases protein expression related to the cell cycle, promoting EC migration and proliferation (164). The effect of E2 on RV capillary vascularization and RV ERK1/2 activation is only weakened in non-selective ER retardation, and is not effective in ERα or ERβ selective arrest (90). Estrogen binds to EREs by forming an estrogen-ER complex and regulates VEGF gene transcription (165). E2 enhances the mobilization of endothelial progenitor cells from bone matrix, and promotes its entry into sites of neovascularization through eNOS-mediated matrix metalloproteinase-9 expression enhancement in the bone matrix (166). Therefore, E2 may lead to improvements in cardiopulmonary structure and function by increasing myocardial blood vessels and angiogenesis, which may represent an additional mechanism to alleviate PH. This is supported by data showing that E2 cannot rescue PH in the presence of the angiogenesis inhibitor, TNP (12).

### Vasodilation

Exogenous E2 rapidly reduces PA vascular reactivity and acute hypoxic pulmonary vasoconstriction (HPV) in a dose-dependent fashion (167). Changes in endogenous E2 abundance in females can also affect the vasoconstrictor response (168). Recent research suggests that low levels of NO in PAH may not be solely due to reduced NOS expression, but rather, may be influenced by factors that regulate NOS activity (169). The beneficial effects of estrogen in PAH partly reflect its effects on eNOS induction *via* both expression and activation, resulting in NO production, improved pulmonary hemodynamics and vascular remodeling. Although this is likely and predominantly mediated by ERs at the level of gene transcription, estrogen also appears to have non-genomic effects on ERα-mediated eNOS activity in ECs. The combination of these genomic and non-genomic pathways is critical to the vascular protective properties of estrogen (121, 170).

Lahm et al. showed that even physiological changes in endogenous estrogen, such as the menstrual cycle, can affect pulmonary artery vasoreactivity and pulmonary vasoconstriction during acute hypoxia (168). Chronic E2 treatment *in vivo* increases the expression of eNOS, resulting in enhanced endothelium-dependent dilation in aorta and lungs (171). Long-term E2 infusion causes abnormal vasodilation patterns in the lungs of fetal sheep (172). The pulsatile load of the mechanical proximal PA is reduced after E2 supplementation, thereby improving ventricular-vascular coupling (173). High-dose exogenous E2 acutely attenuates PA vasoreactivity and acute HPV in a rapid and dose-dependent manner (167). This immediate effect suggests the role of non-genomic estrogen mechanisms. E2 also rapidly activates eNOS through Src kinase in human EC, inducing the formation of a complex containing ERs, c-Src and p85 (the regulatory subunit of PI3K). The formation of this complex leads to the continuous activation of PI3K, Akt, and eNOS, thereby enhancing the release of NO *via* eNOS (174). NO induced post-translational protein modification protects blood vessels through S-nitrosated cell proteins (175, 176). Indeed, physiologically relevant doses of E2 has been shown to increase protein S-nitrosation in vascular EC through ERα and eNOS. Although both ERα and ERβ specific agonists increase the expression of eNOS protein, only ERα specific agonists activate eNOS through phosphorylation (177). To sum up, E2 increases the protein level of eNOS through the genomic pathway, and activates eNOS in a rapid non-genomic manner in vascular. A similar effect of estrogen on eNOS was also observed in the heart. E2 stimulated the expression of iNOS and eNOS in cardiomyocytes (178). Both, ERα and ERβ, reduce PA vasoconstriction, and the contribution of specific ERs seems to be stimulus specific. ERα appears to mainly regulate phenylephrine-induced vasoconstriction, while ERβ was shown to inhibits HPV (123). ERα also affects women's vascular endothelial function by affecting protein levels and activating eNOS (80). While the anti-atherosclerotic effect of estrogen may be partially mediated by ERα-induced up-regulation of eNOS gene expression and maintenance of EC function and integrity (179).

### Immunomodulation

A large body of evidence shows that inflammation and autoimmunity play important roles in the pathogenesis of PAH. At the beginning of the disease, the inflammatory response, caused by injured small pulmonary vascular endothelium, is believed to protect the body. However, as the disease progresses, Dorfmüller proposed that severe PH results in immune system disorders (180). This ineffective immune regulation (immune imbalance) may be related to the pathology and biology of PAH and leads to aggravation of the disease (181). In and around the reconstructed lung resistance vessel wall and near the plexiform lesions of PAH patients and PH animal models, varying degrees of perivascular inflammatory infiltration have been found, including T lymphocytes and B lymphocytes, macrophages, dendritic cells and mast cells (182), and elevated levels of proinflammatory cytokines (such as IL-1β and IL-6) in serum (183). Perivascular inflammation plays an important role in the process of vascular remodeling (184). Pulmonary vascular remodeling in HPH requires the recruitment of circulating mesenchymal precursors of the monocyte/macrophage lineage (185), which is essential for the late onset of HPH (186). Studies investigating links between estrogen and inflammation in other diseases have revealed that proinflammatory cytokines such as TNFα, IL-1 and IL-6 stimulate the activity of fibroblast aromatase and increase the level of estrogen (187, 188). Up-regulation of CYP1B1 leads to increased estrogen catabolism to 4-OHE and 16α-OHE1, which have significant angiogenic, pro-inflammatory and mitogenic properties, and thus promote the development of PAH (46). However, estrogen-induced PAH recovery is related to the inhibition of inflammation, and E2 limits the infiltration of lung monocytes/macrophages associated with PAH (12). E2 mediated inhibition of inflammation in the mouse lung is associated with modified levels of vascular cell adhesion molecules and proinflammatory mediators (189). Further, E2 has been shown to regulate several genes involved in inflammation during hypoxia in an ER-dependent manner. The anti-inflammatory effect of E2 appears to be predominantly mediated by ERβ (190, 191).

Another metabolite of estrogen, 2-ME2, also suppresses inflammation in PAH. 2-ME has significant anti-inflammatory and immunomodulatory effects (192). In several cardiovascular and kidney injury models, this effect is manifested by inhibiting macrophage influx/activation (193). Moreover, contrary to 16α-OHE1, 2-ME2 is found to inhibit the expression of inflammatory cytokines (TNF-α, IL-6 and PGE2) in other disease models, reducing local inflammation and preventing new angiogenesis (192). By blocking inflammatory cytokines, 2-ME reduces its stimulation of aromatase activity (194), which in turn may reduce E2 levels in local tissues. E2 appears to regulate lung inflammation in a sex-independent and age-restricted manner *via* ERα (195). Although the specific effect of inflammation on E2 metabolism in PAH is unclear, spontaneous inflammation is known to increase the production and utilization of E2 and its proinflammatory metabolites, which influences the pathology of severe PAH.

## Estrogen and Pregnancy in PAH

Pregnancy further complicates this complex estrogen signaling process. PAH is considered a significant risk factor during pregnancy (196). The levels of progesterone and estrogen continue to increase significantly throughout pregnancy (197–200). Other sex hormones such as DHEA and testosterone also increase during pregnancy (198, 201). These hormones mediate vasodilation (123, 167, 168, 202–204), considered a major driver for the increase in plasma volume caused by pregnancy (204, 205). In addition, elevated E2 and DHEA during pregnancy are thought to have a beneficial effect on RV function (206, 207). However, during pregnancy, especially during the perinatal period, the increase in RV pressure caused by hemodynamic changes and volume changes may far exceed the protective effects mediated by E2 and DHEA, resulting in RV failure. Moreover, while estrogen and some of its metabolites can promote pulmonary vascular remodeling in PAH (14, 18, 46), it remains uncertain whether the worsening of PAH observed in patients after pregnancy is due to, in part, to the direct impact of sex hormones on the cardiopulmonary system. Rapid deterioration of PAH often occurs in the postpartum period (208, 209), when levels of sex hormones drop sharply (210). The protective effects of sex hormones on PAH are likely reduced as their levels decrease, which at least partly contributes to postpartum RV failure. Broadly, the increase in sex hormones caused by pregnancy and their sharp decline during postpartum have complex effects on the pulmonary vascular system and RV in PAH. There are a paucity of studies in this field with significant need to fill this gap.

## Conclusion

Although significant progress has been made in our understanding of estrogen synthesis and catabolism, earlier research efforts primarily focused on circulating levels of estrogen and/or its metabolism in the liver. Measuring circulating estrogen concentration or its concentration in urine, however, may not provide an adequate window into comprehensively studying the role of estrogen biology in PAH. A better understanding of the local concentrations of estrogen and its metabolites in the heart and lungs, enzymes involved in estrogen metabolism, and their activity, is required to further clarify the role of estrogen in PAH.

Beyond estrogen, testosterone- as a precursor of estradiol- also deserves consideration when discussing mechanisms of sex differences. Studies show that testosterone, largely, has an anti-inflammatory effect, resulting in more pronounced pulmonary artery relaxation than estrogen (202, 203, 211). However, observations from rodent PH models, where female rodents with ovariectomy demonstrate greater severity of disease than male rats with female rats considered the least severe, suggest that the overall contribution of testosterone in the development of PAH may not be as significant as estrogen. In contrast, E2 metabolism in PAH has also begun to be better characterized. In general, increased 16α-OHE1 appears to promote the development of PAH, while 2-ME2 likely contributes to reversal of disease-related phenotypes. E2 appears to be protective in the RV but its role in the pulmonary circulation is less clear. Inhibition of the major estrogen metabolizing enzymes, aromatase (estrogen synthesizing enzyme) and CYP1B1 (catalyzes the catabolism of estrogen to “PH-damaging” metabolites), represent novel potential therapeutic targets in PAH. However, factors that influence estrogen synthesis and catabolism remain poorly characterized in PAH. For example, can increased production of 2-ME2 promote a therapeutic role in PAH? Estrogen and its metabolites play diverging roles in different cells, and in particular cases the same cells. Comprehensive studies of their activity in various cells related to PAH, remain essential for increasing our understanding of the role of estrogen in PAH and these are still lacking.

Finally, establishing reproducible and translatable observations from animal models of PAH remains a top priority. For studies that focus on understanding the “estrogen paradox,” selecting an animal model that better mirrors features of the human phenotype is critical. Importantly, recognizing that the estrous cycle and hormone levels of commonly used rats and mice are not similar to those of human patients is equally as important. Moreover, there is a significant knowledge gap in the changes across the full spectrum of estrogen synthesis, catabolism and signaling in PAH animal models during disease development, further highlighting limitations of animal models. Although none of the animal models can fully mimic the phenotype of patients with PAH, researchers should methodically select animal model(s) that are most suitable for the hypothesis and aims of the study.

Future studies will need to address a variety of topics in this field including: (1) changes in estrogen and its metabolic activity in patients and animal models during the progression of PAH; (2) the role of estrogen and its metabolites in a tissue- and temporal-specific manner in PAH; (3) defining the spectrum of estrogen synthesis-enzymes and their contributions to the estrogen content in the blood. (4) the distribution and ratio of ERα, ERβ, and GPER in cardiopulmonary tissues and their function, as well as changes in their expression and modifications with age and PAH development; (5) transcriptional programming regulated by ERα, ERβ, or/and GPER as well as estrogen metabolites; (6) the bi-directional relationship between estrogen signaling and the presence/progression of disease including elucidating any potential positive or negative feedback loops.

In conclusion, estrogen influences cardiopulmonary function in health and disease in several ways. Differences in estrogen-related enzyme activity and estrogen signaling can be observed in normal and PAH lungs, indicating that there is a correlation between PAH and estrogen catabolism, metabolism and signaling. The functions and activities of specific receptors, the concentration and ratio of estrogen and its metabolites, as well as their local concentration relative to the circulatory system, the interaction between various sex hormones, age, comorbidity, genetics, and other factors are involved in the relationship between PAH and estrogen. By analyzing and comparing existing studies, the joint contribution of specific ERs or signaling pathways and specific metabolites may partially clarify the controversy about the role of estrogen in PAH. The knowledge gained from these future investigations will provide the necessary understanding on mechanisms and better prepare us to tackle the sex gap in PAH. Ultimately, it is hoped that these studies will fuel the discovery of novel therapeutic targets for this uncurable and devastating disease.

## Author Contributions

YS, HT, SB, and AD wrote the manuscript with input from SS, QG, and JW. All authors contributed to the article and approved the submitted version.

## Conflict of Interest

The authors declare that the research was conducted in the absence of any commercial or financial relationships that could be construed as a potential conflict of interest.

## Publisher's Note

All claims expressed in this article are solely those of the authors and do not necessarily represent those of their affiliated organizations, or those of the publisher, the editors and the reviewers. Any product that may be evaluated in this article, or claim that may be made by its manufacturer, is not guaranteed or endorsed by the publisher.
